# Immune system-related plasma extracellular vesicles in healthy aging

**DOI:** 10.3389/fimmu.2024.1355380

**Published:** 2024-04-03

**Authors:** Xin Zhang, Sisi Ma, Janet L. Huebner, Syeda Iffat Naz, Noor Alnemer, Erik J. Soderblom, Constantin Aliferis, Virginia Byers Kraus

**Affiliations:** ^1^ Duke Molecular Physiology Institute, Duke University School of Medicine, Duke University, Durham, NC, United States; ^2^ Department of Orthopaedic Surgery, Duke University School of Medicine, Duke University, Durham, NC, United States; ^3^ Institute for Health Informatics, University of Minnesota School of Medicine, Minneapolis, MN, United States; ^4^ Duke Proteomics and Metabolomics Core Facility, Duke University School of Medicine, Duke University, Durham, NC, United States; ^5^ Department of Medicine, Duke University School of Medicine, Duke University, Durham, NC, United States

**Keywords:** extracellular vesicles, healthy aging, immune system, cytokines, proteomics, surface markers, proliferation

## Abstract

**Objectives:**

To identify age-related plasma extracellular vehicle (EVs) phenotypes in healthy adults.

**Methods:**

EV proteomics by high-resolution mass spectrometry to evaluate EV protein stability and discover age-associated EV proteins (n=4 with 4 serial freeze-thaws each); validation by high-resolution flow cytometry and EV cytokine quantification by multiplex ELISA (n=28 healthy donors, aged 18-83 years); quantification of WI-38 fibroblast cell proliferation response to co-culture with PKH67-labeled young and old plasma EVs. The EV samples from these plasma specimens were previously characterized for bilayer structure, intra-vesicle mitochondria and cytokines, and hematopoietic cell-related surface markers.

**Results:**

Compared with matched exo-EVs (EV-depleted supernatants), endo-EVs (EV-associated) had higher mean TNF-α and IL-27, lower mean IL-6, IL-11, IFN-γ, and IL-17A/F, and similar mean IL-1β, IL-21, and IL-22 concentrations. Some endo-EV and exo-EV cytokine concentrations were correlated, including TNF-α, IL-27, IL-6, IL-1β, and IFN-γ, but not IL-11, IL-17A/F, IL-21 or IL-22. Endo-EV IFN-γ and exo-EV IL-17A/F and IL-21 declined with age. By proteomics and confirmed by flow cytometry, we identified age-associated decline of fibrinogen (FGA, FGB and FGG) in EVs. Age-related EV proteins indicated predominant origins in the liver and innate immune system. WI-38 cells (>95%) internalized similar amounts of young and old plasma EVs, but cells that internalized PKH67-EVs, particularly young EVs, underwent significantly greater cell proliferation.

**Conclusion:**

Endo-EV and exo-EV cytokines function as different biomarkers. The observed healthy aging EV phenotype reflected a downregulation of EV fibrinogen subpopulations consistent with the absence of a pro-coagulant and pro-inflammatory condition common with age-related disease.

## Introduction

Immune system aging has been associated with a phenotype termed immunosenescence that is characterized by a decreased number and deficient function of all major immune cell subsets (such as T cells, B cells, neutrophils, natural killer cells, monocytes, macrophages, and dendritic cells) (1–4). Stress factors, such as bone fracture, accentuate immunosenescence (1). Our recent causal analyses of a large human cohort of older adults (n=1507) identified lymphocytes as one of the key potentiators of longevity, and provide a strong rationale for further studies of aging-related changes in the immune system (5).

Extracellular vesicles (EVs), nanosized particles that are secreted by almost all mammalian cells, carry surface markers and biological effectors (including mitochondria, small non-coding RNAs such as microRNAs and piRNAs, mRNAs, proteins, cytokines) from their parent cells, reflecting the health status of the body, aging and involvement in age-related diseases (1, 6–9). EVs are reported to play crucial roles in intercellular communication and regulation of immune function through the transport of their cargo to recipient cells, in some cases mediated by surface ligand-receptor interactions (9–13). To avoid lysosomal degradation following internalization by recipient cells, EVs must fuse with the plasma membrane or the endosome of the recipient cells. This fusion step leads to the integration of membrane proteins and lipids of the EVs into the plasma membranes or endomembranes of the recipient cells; as a result, the cargo effectors of the EVs are released into the cytosol of the recipient cells (14). For instance, EVs carry intact and functional mRNAs that can be transferred to recipient cells where they are translated into proteins (13). MHC class I and II molecules on the surface of B cell-derived EVs can present antigens to induce proliferation of recipient T cells (12).

Understanding EV biomarker alterations in healthy aging may help to capture early signs of abnormal alterations or pathological aging that may be involved in the initiation and development of age-related diseases. Our previous study reported age-associated declines in healthy donors of several plasma EV subpopulations related to immune cells, along with their mitochondrial cargo (6). Similarly, we observed an immunosenescence phenotype in aged mice, exacerbated by the physical stressor of fracture injury in old but not young mice, characterized by age-dependent declines in multiple types of circulating immune cells and small EVs related to T lymphocytes, B lymphocytes and neutrophils (1). In the present study, we aimed to identify changes in additional immune system-related plasma EV biomarkers in healthy aging, using the EV samples from plasma specimens of healthy donors that were previously characterized for bilayer structure, intra-vesicle mitochondria and cytokines, and hematopoietic cell-related surface markers using high-resolution flow cytometry (6).

## Methods

### Study participants

A total of 28 EDTA plasma specimens were analyzed in this study: (1) 12 healthy donors (mean age 40 ± 18 years, range 18-78; mean BMI 26 ± 6 kg/m^2^; 50% male) from a commercial vendor (Zen-bio acquired with IRB approval); and (2) 16 healthy donors (age 68 ± 8 years, range 55-83 years; mean BMI 27 ± 3 kg/m^2^; 50% male) from the completed Genetics of Generalized Osteoarthritis (GOGO) study (15) with informed consent under IRB approval of Duke University. All samples were frozen at -80°C until analysis. EVs from healthy donors aged <35 years (n=6) and >45 years (n=22) were defined as ‘young’ EVs and ‘old’ EVs, respectively. Multiplex ELISA and high-resolution multicolor flow cytometry used EVs from all 28 healthy donors, while mass spectrometry-based proteomic analysis was performed on EVs isolated from 4 of these healthy donors from Zen-bio (aged 18 years old [yo] female [F], 31 yo male [M], 52 yo F, and 78 yo M; BMI 25.6, 25, 27.4, and 23.4 kg/m^2^).

### EV separation and characterization

Following the protocols presented in our previous publications (6, 7), plasma was isolated from blood specimens collected in EDTA tubes by centrifugation (Relative Centrifugal Force 1600-2000g) for 10 mins at room temperature or 15 mins at 4°C to remove pellets of cells and debris; samples were aliquoted and frozen at −80°C until analysis. On the day of EV separation, all frozen plasma was completely thawed and centrifuged at 2000 g for 10 minutes at 4°C to remove any remaining debris. EV pellets and EV-depleted supernatants were separated from plasma by polymer-based precipitation (ExoQuick, System Biosciences). The EV samples from these plasma specimens (6) have been previously validated to have a bilayer structure and size diversity ranging from small (<100nm), to medium (100-1000nm), and large (up to 6000nm), and carry mitochondria, traditional EV markers (CD81, CD9, CD63, CD29), and hematopoietic cell-related markers (CD4, CD8, CD56, CD15, CD14, CD68, CD19, CD235a, CD41a, CD31, CD34, HLA-ABC, HLA-G and HLA-DRDPDQ). Platelet-related EVs were rare in the plasma EVs from this study cohort; the mean percentages in LEV, MEV and SEV were 0.8%, 0.04% and 0% for CD61^+^CD41a^+^ vesicles, 1.3%, 0.2% and 0.08% for CD61^+^CD41a^−^ vesicles, and 3.0%, 1.1% and 0.05% for CD61^−^CD41a^+^ vesicles.

### Multiplex ELISA

Following the protocols previously described (7), EV pellets were lysed in NP40 lysis buffer (Thermo Fisher Scientific) in the same volume as the EV-depleted supernatants. The concentrations of endo-EV (associated within or on EVs) and exo-EV (remaining in EV-depleted supernatants) cytokines were measured by multiplex ELISA using the Custom Pro-inflammatory Panel (Panel 1: IL-1β, IL-6, and TNF-α, IFN-γ; Panel 2: IL-27, IL-11, IL-17A/F, IL-21, and IL-22, Meso Scale Diagnostics [MSD]) following the manufacturer’s instructions (7). Notably, for these particular MSD multiplex ELISA kits, “Diluent 2” (containing 0.01-0.02% detergent, most often Triton X per communication with supplier) was used to dilute standards and samples.

### Mass Spectrometry-based proteomics

EV pellets were isolated from 50 µl plasma at baseline (first thaw) and after serial thaws of 1, 3, and 6 times, then were treated with 100 µl of 8 M urea. EV pellets were probe-sonicated and processed for proteomics by high-resolution mass spectrometry coupled with label-free quantification using a nanoAcquity UPLC system (Waters Corp) coupled to an Orbitrap Fusion Lumos high-resolution accurate mass tandem mass spectrometer equipped with a FAIMS Pro system (ThermoFisher Scientific) by the Duke Proteomics and Metabolomics Core Facility; sample preparation and proteomic analyses were performed as described previously (16). Peptide amino acid designations were assigned based on the SwissProt Human database (downloaded Nov 2019). To correlate volumetric measures from flow cytometry to proteomic measures, the relative quantitative expression value of each peptide (gravimetric (per ug) basis) was converted to a volumetric measure (per µl) by dividing the peptide value by the volume taken for 20 µg EV-derived protein in each sample that was used for sample preparation. In addition, the peptide values for the measurements from plasma-derived EVs have been multiplied by 2 to account for the 2-fold dilution of the plasma-derived EVs in 8 M urea. The normalized data were used for statistical analyses. STRING network analyses (17) were performed to analyze protein interactions and functional enrichment of the proteins comprised of the identified plasma EV peptides.

### High-resolution multicolor flow cytometry

EVs from 20 µl plasma were utilized for flow cytometry-based profiling. EV pellets were resuspended in double-filtered PBS (df-PBS) by 100 nm filters (MilliporeSigma) and stained with fluorescence-conjugated antibodies against human fibrinogen alpha chain (FGA), fibrinogen beta chain (FGB), and fibrinogen gamma chain (FGG) (Novus Biologicals). The high-resolution Sony MA 900 Multi-Application Sorter (Sony Biotechnology) was configured to ensure acquisition events of df-PBS <10 events/second; relative size distribution of EVs were estimated using reference beads with mean size 100, 1000 and 6000 nm (Bangs Laboratories) (6). The fluorescence background was determined using unstained and antibody-stained EVs and UltraComp™ eBeads Plus (ThermoFisher Scientific). The percentages (%) of plasma EVs carrying each surface marker were determined using flow cytometry; flow cytometric data analysis was performed using FCS Express 5 software (*De Novo* Software).

### PKH67 pre-labeling

EVs separated from the plasma of healthy donors were resuspended in staining buffer and stained using the PKH67 Green Fluorescent Cell Linker Kit (MilliporeSigma), used to label cell membranes, for 20 minutes at room temperature in the dark, shaking at 300 rpm; the No EV Control consisted of staining buffer with PKH67 but without EVs (6). After pre-labeling, plasma EVs and the No EV Control were re-pelleted by polymer-based precipitation; unbound PKH67 remaining in supernatants was removed. The EV pellets and No EV Control were gently washed twice with df-PBS to remove any residual unbound PKH67, then resuspended in df-PBS for coculture with WI-38 cells.

### WI-38 cell culture and treatment

The WI-38 human diploid fibroblast cell line (American Type Culture Collection, ATCC® CCL-75™) was cultured starting from passage (PA)12 in normal culture medium consisting of Minimum Essential Medium (Gibco) supplemented with 10% heat-inactivated Fetal Bovine Serum (Gibco) and L-Glutamine–Penicillin–Streptomycin solution (MilliporeSigma) as we previously reported (18). Trypsin was used between each passage; PA16-PA23 were used for the experiments. To test the effects of EVs, WI-38 cells were cultured in 1 ml/well Opti-MEM reduced serum medium (ThermoFisher Scientific) with penicillin-streptomycin (25.0 ug/ml, MP Biomedical) in the absence or presence of PKH67-labeled plasma EVs (derived from 100 µl plasma) and the No EV control for immunofluorescence imaging and EdU (5-ethynyl-2´-deoxyuridine) proliferation assay.

### Immunofluorescence imaging

After a 2-hour coculture of WI-38 cells with PKH67-labeled plasma EVs (derived from 100 µl plasma) and the No EV control, the culture medium was discarded, and the cells on the plate were washed twice with 500 µl HBSS (Gibco). The cells were fixed with 100 µl 4% PFA for 15 minutes at 37°C, then washed three times with HBSS. WI-38 cell membranes were stained with Wheat Germ Agglutinin (WGA, ThermoFisher Scientific) and washed twice with HBSS. The cells were permeabilized with 0.2% Triton-X-100 (MilliporeSigma) for 5 minutes, followed by cell nuclei staining with DAPI (MilliporeSigma). The cells were washed twice with HBSS. Immunofluorescence images were captured on an Olympus IX70 Inverted Phase Contrast DIC Fluorescence Microscope accompanied with X-Cite 120LED Boost High-Power LED illumination System (EXCELITAS Technologies) and analyzed with cellSens Standard Software (Olympus).

### EdU proliferation assay

**F**ollowing the previously reported protocol (18), WI-38 cells were cultured with EdU for 24 hours, and cell proliferation was detected using a Click-iT™ EdU Alexa Fluor™ 647 Flow Cytometry Assay Kit (Thermo Fisher Scientific) following the manufacturer’s instructions. Cells without exposure to EdU were used to determine the fluorescence background. The percentages (%) of cells expressing each tested marker were determined using a high-resolution multicolor Attune NxT Flow Cytometer (ThermoFisher Scientific), and data analysis was performed using FCS Express 5 software.

### Statistical analyses

Analyses performed in this study included the following statistical methods. (1) Shapiro-Wilk test was used to assess the data distribution. (2) Comparisons of mean concentrations between endo-EV and exo-EV cytokines were performed using Wilcoxon matched-pairs signed rank test and Spearman correlations were used to assess the associations of the endo-EV cytokine concentrations with the corresponding exo-EV cytokine concentrations. (3) The proteomic data for 4 individuals (4 serial freeze-thaw samples [at baseline (first thaw) and after serial thaws of 1, 3, and 6 times] for each individual, totaling 16 EV samples) were used to assess the freeze-thaw effects using Wilcoxon matched-pairs signed rank test. (4) Mixed models were used to evaluate the association of EV peptides with age using proteomic data from the 16 EV samples; to account for the fact that each individual participant had 4 serial freeze-thaw samples, individual participants were modeled as the random effect. We fitted random intercept models for each peptide where the peptide was the dependent variable, and age the fixed effect. Some peptides were missing measurements; we only fitted models for peptides with at least one measurement available for each participant. (5) We validated associations of age with the percentage of FGA^+^, FGB^+^ and FGG^+^ plasma EV subpopulations quantified by flow cytometry using Spearman correlations (n=28). (6) Comparisons among the percentages of FGA^+^, FGB^+^ and FGG^+^ plasma EV subpopulations profiled by flow cytometry were performed using the Friedman test with Benjamini-Hochberg correction for multiple comparisons. (7) Comparisons between WI-38 cells cultured in the absence or presence of young and old plasma EVs were assessed by repeated measures ANOVA with Benjamini-Hochberg correction for multiple comparisons. (8) Comparisons between percentages of EdU^+^ proliferating cells in gated PKH67-EVs^+^ and PKH67-EVs− cells were assessed by paired t-test. Statistical significance was defined as p<0.05 or FDR * q<0.05 as described in each figure legend.

## Results

### Some cytokines were enriched in plasma endo-EVs

**Figure 1:**
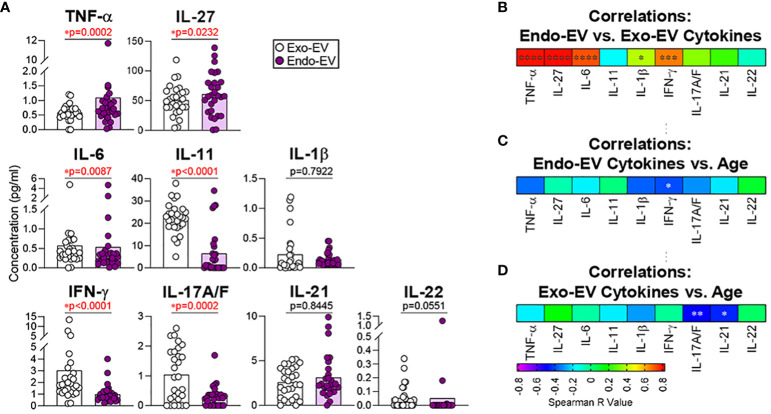
The mean concentrations of TNF-α and IL-27 were significantly higher in EVs (endo-EV) than the corresponding EV-depleted supernatants (exo-EV) of plasma from healthy donors. The concentrations of exo-EV and endo-EV cytokines in plasma from healthy donors (n=28) were measured by multiplex ELISA. **(A)** The graphs represent the summary concentrations of endo-EV and exo-EV cytokines. Comparisons were performed using Wilcoxon matched-pairs signed rank test; significant results were defined as * p <0.05. **(B)** Spearman correlations were used to assess associations of the endo-EV cytokine concentrations with the corresponding exo-EV cytokine concentrations. **(C, D)** Spearman correlations were used to assess associations of age with the concentrations of the indicated endo-EV **(C)** and exo-EV **(D)** cytokines. Heat maps depict the Spearman correlation coefficient r values; significant results were indicated as * p <0.05. **p <0.01, ***p <0.001 and ****p <0.0001.  .

We previously reported that the plasma concentrations of endo-EV TNF-α and IFN-γ, but not the corresponding exo-EV cytokines, were associated with knee radiographic osteoarthritis progression (19), indicating that endo-EV and exo-EV cytokines should be considered different biomarkers and evaluated separately. This prompted us to look at both endo- and exo-EV cytokines separately in healthy controls. Compared to the exo-EV compartment, mean concentrations of endo-EV TNF-α and IL-27 were significantly higher, but IL-6, IL-11, IFN-γ, and IL-17A/F significantly lower (**Figure 1A**). There were no statistically significant differences in endo-EV and exo-EV mean concentrations of IL-1β, IL-21, and IL-22 (**Figure 1A**). Endo-EV and exo-EV concentrations of TNF-α, IL-27, IL-6, IL-1β, and IFN-γ, but not IL-11, IL-17A/F, IL-21 and IL-22 were significantly correlated (**Figure 1B**). Endo-EV IFN-γ concentrations (**Figure 1C**) and exo-EV IL-17A/F and IL-21 concentrations (**Figure 1D**) were significantly negatively associated with age. Concentrations of TNF-α and IFN-γ in total (unprocessed) plasma (measured independently), were similar to the sum of their concentrations from endo-EVs and exo-EVs; total (unprocessed) plasma concentrations of IL-6 and IL-1β were only slightly lower than the sum of their concentrations from endo-EVs and exo-EVs (**Supplementary Figure 1A**), suggesting there was minimal loss or degradation of cytokines by the EV separation procedure.

Pretreatment of EVs with NP-40 released small additional amounts of IL-6 and IL-1β from EVs (**Supplementary Figure 1B**). MSD ELISA methodology detected the majority of endo-EV cytokines with the use of the standard buffer (Triton-X containing) without the further addition of NP40; concentrations yielded without additional NP40 were a mean 91.9%, 78.1%, 60.1%, and 72.0% of concentrations for TNF-α, IL-6, IL-1β, and IFN-γ, respectively, yielded with additional NP40 (**Supplementary Figure 1A**).

### EVs protected peptide cargo from degradation or loss during repeated freeze-thaw cycles

Several previous studies reported that repeated freeze-thaw cycles do not affect the stability of RNAs and miRNAs in EVs from human plasma and urine specimens (20–22). In this study, based on a high throughput and non-targeted proteomic analysis, we identified 7388 peptides in plasma EVs from healthy donors. We compared the relative quantitative expression of endo-EV peptides at baseline (first thaw) and after 1, 3, and 6 additional serial freeze-thaw cycles. We confirmed the stability of EV proteins, reflected in the lack of significant change (p value ranges 0.1003-1, all adjusted p=1, **Supplementary Table 1**) in peptide concentrations with repeated freeze-thaw cycles.

### Fibrinogen-related EV peptides were negatively associated with healthy aging

Using high-resolution mass spectrometry, we identified 7388 peptides (corresponding to 605 proteins) in plasma EVs of this healthy donor cohort. Using mixed models, we identified 4 peptides that were significantly (p<0.05) and negatively associated with healthy ageing, including: fibrinogen beta chain (FGB, P02675 [53–72]); immunoglobulin lambda constant 1 (IGLC1, B9A064 [113–131], P0CG04 [5–23], P0DOX8 [115–133]); Vitamin D-binding protein (GC, P02774 [181–203]); and Apolipoprotein B-100 (APOB, P04114 [455–490]) (**Supplementary Table 1**). In addition, two EV peptides from the fibrinogen alpha chain (FGA) and two from the fibrinogen gamma chain (FGG) exhibited a negatively associated trend with age (p values ranged 0.0723-0.0985, **Supplementary Table 1**). None of the fibrinogen-related plasma EV peptides were significantly positively associated with age (**Supplementary Table 1**). Fibrinogen is predominantly but not exclusively produced by hepatocytes in the liver. Fibrinogen is comprised of three pairs of polypeptide chains, Aα, Bβ and γ that are encoded by genes *FGA*, *FGB* and *FGG*, respectively (23). In plasma of healthy donors, we identified 107, 86 and 66 peptides from FGA, FGB and FGG, respectively (**Supplementary Table 2**).

### FGA^+^, FGB^+^ and FGG^+^ plasma EV subpopulations declined with age

**Figure 2:**
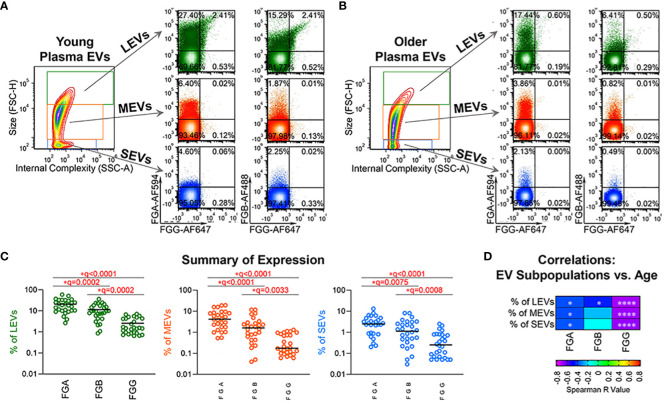
Fibrinogen EV subpopulations, FGA^+^, FGB^+^ and FGG^+^ in plasma, declined with age. **(A–C)** Plasma EVs from healthy donors (n=28) were profiled for FGA, FGB and FGG expression by high-resolution flow cytometry. Representative plots from plasma EVs of young (**A**, 19 year old, male) and old (**B**, 66 year old, male) healthy donors: the representative contour plot presents size (FSC-H: Forward Scatter-Height) and granularity (SSC-A: Side Scatter-Area) of large (LEVs, 1000-6000 nm), medium (MEVs, 100-1000 nm) and small (SEVs <100 nm) EVs; the relative size distribution was estimated by size reference beads; the representative color dot plots display the tested surface markers in the gated LEVs, MEVs and SEVs. The scatterplots represent the percentage of FGA^+^, FGB^+^ and FGG^+^ plasma EV subpopulations **(C)**. Comparisons were performed using Friedman test with Benjamini and Hochberg multiple comparisons; significant results were defined as FDR value * q <0.05. **(D)** The heatmap depicts results of Spearman correlations (r values) used to assess associations of age with the percentage of the indicated EV subpopulations in this healthy donor cohort (n=28); significant results are indicated as * p <0.05, and **** p <0.0001.

In our recent publication, we identified FGA/FGB/FGG EV peptides associated with disease severity of knee osteoarthritis, a disease related to pathological aging (16). To validate the age association of plasma EVs bearing fibrinogen on their surface in healthy aging, we next used high-resolution flow cytometry to verify the presence and quantify the amount of FGA^+^, FGB^+^ and FGG^+^ EV subpopulations in plasma samples of healthy donors (n=28). FGA, FGB and FGG were detected on the surface of plasma EVs of all sizes, with a wide range of frequencies (**Figures 2A–C**). The frequency of FGA^+^ EV subpopulations was the highest; the frequency of FGG^+^ EV subpopulations was the lowest (**Figure 2C**), which was consistent with the number of corresponding peptides in plasma EVs (**Supplementary Table 2**). The percentage of FGA^+^ and FGG^+^ EV subpopulations of all sizes, and FGB^+^ large EVs were significantly negatively associated with age (**Figure 2D**).

### Young plasma EVs significantly induced proliferation of recipient cells

**Figure 3:**
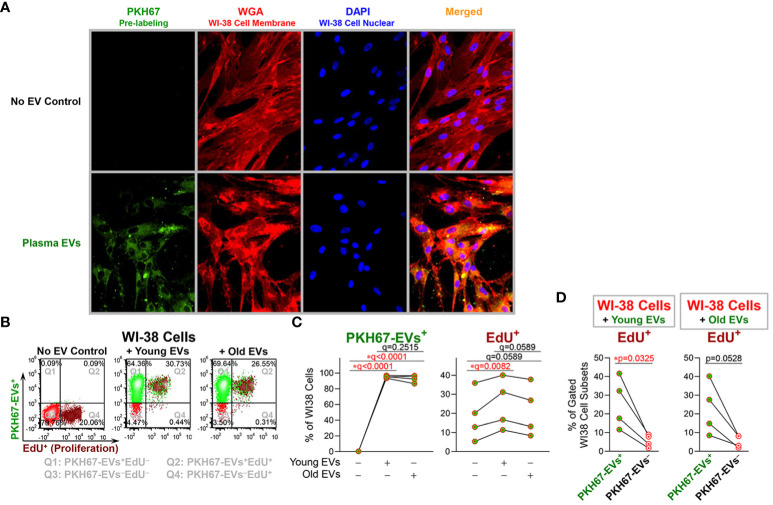
Young plasma EVs significantly induced proliferation of recipient cells. EVs separated from plasma of healthy donors (n=4; age 18, 19, 52, 52; 50% female) were stained with PKH67; the No EV Control consisted of staining buffer with PKH67 but without EVs. After pre-labeling, plasma EVs and the No EV Control were re-pelleted by polymer-based precipitation; unbound PKH65 remaining in supernatants was removed; the EV pellets and No EV Control were gently washed twice with df-PBS to remove residual unbound PKH67, and then resuspended in df-PBS. PHK67-labeled plasma EVs derived from 100 µl plasma and a No EV Control were added to the WI-38 human fibroblasts (passages 16, 17, 18 and 23) cultured in 1ml/well Opti-MEM serum-reduced medium. **(A)** After 2-hours coculture, the culture supernatants were removed, and the cells were fixed and stained with Wheat Germ Agglutinin (WGA) to label WI-38 cell membranes, then permeabilized and stained with DAPI to label WI-38 cell nuclei. Magnification: 20X lens and 2X software setting. **(B–D)** The cells were incubated with EdU for 24 hours. The percentages of WI-38 cells with internalized EVs (indicated by PKH67^+^ signal), and proliferating (indicated by EdU^+^ signal) were determined by flow cytometry. The color dot plots display the representative staining of WI-38 cells **(B)** with each quadrant depicting different cell staining; Q1 (top left): PKH67-EVs^+^EdU−, Q2 (top right): PKH67-EVs^+^EdU^+^, Q3 (bottom left): PKH67-EVs−EdU−, and Q4 (bottom right): PKH67-EVs−EdU^+^ cells. The differential percentages of PKH67-EVs^+^ and EdU^+^ cells in all WI-38 cell singlets **(C)** were assessed by repeated measures ANOVA with Benjamini and Hochberg multiple comparisons; statistical significance was defined as FDR * q<0.05. The percentages of EdU^+^ proliferating cells in gated PKH67-EVs^+^ and PKH67-EVs− cells **(D)** were produced by Q2/(Q1+Q2) and Q4/(Q3+Q4), respectively; the differences were assessed using paired t-test; statistical significance was defined as * p<0.05.

To test the effects of age-related alterations of plasma EVs on recipient cells, we cocultured PKH67-labeled plasma EVs with WI-38 fibroblast cells. By two hours after coculture, PKH67-labeled plasma EVs were internalized by WI-38 cells as indicated by green fluorescence in the cytoplasm of WI-38 cells cultured with PKH67-labeled EVs but not the No EV control (**Figure 3A**). 24 hours after coculture, flow cytometry identified that over 95% of WI-38 cells contained PKH67-labeled plasma EVs (**Figures 3B, C**), and there was no statistically significant difference in the percentage of recipient WI-38 cells that internalized PHK67^+^ young and old plasma EVs (**Figure 3C**). Compared to the No EV control, WI-38 cells cocultured with young plasma EVs exhibited a significantly higher proliferation activity as indicated by EdU^+^ signals; a similar induction of proliferation by old plasma EVs was observed but the result did not pass the threshold for statistical significance (**Figure 3C**). The induction of recipient WI-38 cell proliferation by young plasma EVs was consistently higher than induction by old plasma EVs, but the difference between young and old did not pass the threshold for statistical significance (**Figure 3C**).

Furthermore, the percentage of EdU^+^ proliferating WI-38 cells was significantly higher for the PKH67-EVs^+^ cells that internalized EVs, compared with the small number of the PKH67-EVs− cells that did not internalize EVs (**Figure 3D**). In WI-38 cells cocultured with old plasma EVs, we also observed a higher percentage of EdU^+^ proliferating cells in PKH67-EVs^+^ cells than PKH67-EVs− cells, but the difference did not reach statistical significance (**Figure 3D**).

## Discussion

Cytokines in biofluids have been well-documented to play crucial roles in aging, age-related disease and longevity (24–26), and can be carried by EVs that have the capability of delivering cytokines to recipient cells (1, 6–9). Of the cytokines we analyzed in this study by MSD ELISA, in most cases, we could detect similar concentrations associated with EVs (in or on their surface) with and without an additional EV lysis process; we attribute this to Triton X in the sample diluent of MSD ELISA kits, which has been used to lyse EV samples to release cytokines in previous studies (27, 28). We also observed that the cytokine concentrations in (unprocessed) plasma, i.e. plasma without an EV separation procedure, quantified by MSD ELISA, well represent the sum of the concentrations of exo-EV cytokines and all or a major part of endo-EV cytokines.

In this study of healthy donors, some cytokines had higher endo- than exo-EV mean concentrations (TNF-α and IL-27), some lower concentrations of endo- than exo-EV concentrations (IL-6, IL-11, IFN-γ, and IL-17A/F), and some similar concentrations (IL-1β, IL-21, and IL-22). These results are consistent with our prior observation of a higher mean concentration of endo-EV TNF-α than exo-EV TNF-α in plasma of patients with knee osteoarthritis (7). Our findings confirm an abundance of cytokines in EVs; these could potentially contribute to EV-mediated intercellular communication (29). Due to the multiplicity of active constituents, EVs are understood to act in a complex manner (30) and the exact mechanism by which endo-EV cytokines may mediate autocrine, paracrine, or endocrine (distant) effects are unknown. Several cytokines (such as IL-1, TNF-α, IFN-γ, TGF-β, and IL-15) have both membrane-associated forms and soluble forms (31–36). The membrane and transmembrane forms of cytokines in EVs could be envisioned to interact with recipient cell receptors and thereby activate signaling cascades.

Interestingly, the endo-EV concentrations of some cytokines (TNF-α, IL-27, IL-6, IL-1β, and IFN-γ) in plasma of healthy donors were highly associated with the corresponding exo-EV cytokine concentrations determined by ELISA. Similarly, we previously observed significant associations between the endo-EV and exo-EV concentrations of TNF-α, IL-6, and IFN-γ in plasma of patients with knee osteoarthritis, but not IL-1β (7). In older adults, previous studies reported associations of high levels of IL-1, IL-6, TNF-α, and IFN-γ with increased risk of morbidity and mortality (26). In this healthy donor cohort, we observed that the endo-EV concentrations of IFN-γ, the signature cytokine of type 1 T helper (Th1) cells, and the exo-EV concentrations of IL-17A/F and IL-21, the signature cytokines of type 17 T helper (Th17) cells (37), were significantly negatively associated with age, suggesting decreased effector cytokine production and EV-mediated-transport of Th1 and Th17 cytokines in healthy aging. In contrast, concentrations of IL-1β, IL-6 and TNF-α in plasma measured by ELISA were significantly higher in frailty, a syndrome of pathological aging, compared with non-frail older adults (38); in addition, in female older adults, plasma IFN-γ concentration was higher in frailty than non-frailty (38). Similarly, plasma IL-27 and TNF-α were positively correlated with older age, longer hospitalization, and disease severity in Covid-19 patients (39). We previously reported that the plasma endo-EV concentrations of TNF-α and IFN-γ, but not the corresponding exo-EV cytokine concentrations, were associated with knee radiographic progression of osteoarthritis, a common age-related disease (19). Since ELISA detects both exo-EV and endo-EV cytokines in plasma, but biological concentrations of cytokines and accessibility of cytokines can differ greatly in these two compartments, we advise parallel evaluation of the roles of endo-EV and exo-EV cytokines in age-related diseases and conditions.

It has been well-documented that EVs are involved in aging processes through regulating multiple biogenesis pathways, such as cellular senescence, inflammation, tissue regeneration, oxidative stress, metabolism and autophagy (1, 7, 19, 40, 41). We have observed age-associated declines of multiple immune cell-related EV subpopulations in healthy humans and mice, consistent with an immunosenescence phenotype in healthy aging that is reflected in EV subpopulations (1, 6). Moreover, EV biomarkers indicating immunosenescence can be accentuated by common physical stresses in older adults, such as bone fracture and osteoarthritis (1, 7, 19). Fibrinogen is an important component in the immune system, and plays essential roles in multiple biological processes, including but not limited to blood clotting, fibrinolysis, cellular and matrix interactions, wound healing, and inflammation (23). We recently identified that fibrinogen-related EV peptides were associated with knee osteoarthritis severity (16). However, the involvement of fibrinogen-related EVs in healthy aging is largely unknown. In the present study, we identified a FGB peptide in plasma EVs that significantly declined during healthy aging, while FGA and FGG EV peptides also showed a declining trend with age. In addition, we validated an age-related decline of the frequency of FGA^+^, FGB^+^ and FGG^+^ EV subpopulations in plasma of healthy donors. Although hepatocytes are the primary cell source of fibrinogen, fibrinogen is also synthesized in some extra-hepatic tissues including gene expression of FGG *in vivo* in bone marrow, brain, and lung (42). Activation of the coagulation cascade can result in the formation of fibrin associated with exposure of cryptic epitopes that transforms fibrinogen from a non-inflammatory blood factor to a potent activator of innate immunity (43). Given the associations of increased EV fibrinogen with liver disease (44), autoimmunity in humans and mice (45), and its ability to interact with the integrin β of naive macrophages to aggravate the inflammatory and pro-fibrotic effects of macrophages (46), the downregulation of EV fibrinogen that we observed in healthy aging is consistent with the absence of a pro-coagulant and pro-inflammatory condition common with age-related disease.

Plasma EVs, from both young and old donors, were readily internalized by the majority (>95%) of WI-38 cells. Moreover, compared to cells that did not internalize EVs, higher proliferation activity was observed for WI-38 cells that internalized EVs, both young and old (statistically significant increase in proliferation for young EVs). These and our prior (47) results support a therapeutic and regenerative potential of plasma EVs that might be administered as autologous or allogeneic therapy, and indicate that the age-related alterations of plasma EVs during healthy aging impact their capacity for regulating cell proliferation. Alternatively, we cannot rule out the possibility that cells with a lower baseline proliferation rate were less likely to take up EVs or have stronger lysosomal degradation of internalized EVs. Our previous study reported that the majority of plasma EVs from healthy donors carried functional mitochondria, which declined with age in many immune cell-related EV subpopulations (6). Transferring functional mitochondria and other EV effectors, such as specific proteins and small non-coding RNAs, may contribute to the EV-mediated regulation of cell proliferation in recipient cells, which warrants further investigation.

There were several limitations of the present study. Using ELISA, proteomics and flow cytometry, we identified numerous EV biomarkers in healthy aging. However, the samples size for EV proteomics was relatively small. Therefore, we recommend the need for validation of our findings from these discovery experiments in independent, larger sample sets.

In summary, in a healthy donor cohort comparing endo-EV to exo-EV compartments of plasma, we observed higher mean concentrations of TNF-α and IL-27, lower mean concentrations of IL-6, IL-11, IFN-γ, and IL-17A/F, and similar mean concentrations of IL-1β, IL-21, and IL-22. We also observed a positive association between the endo-EV and exo-EV concentrations of individual cytokines, including TNF-α, IL-27, IL-6, IL-1β, and IFN-γ, but not IL-11, IL-17A/F, IL-21 and IL-22. We identified an age-associated decrease in the endo-EV concentrations of IFN-γ and the exo-EV concentrations of IL-17A/F and IL-21. Given that the concentrations of endo-EV and exo-EV cytokines were not always associated with each other, and their associations with age were also different, we consider endo- and exo-EV compartments as different biomarkers that potentially provide unique biomarker information and therefore as worthy of assessment independent of each other. We identified a consistent negative association of fibrinogen-containing EVs with healthy aging. However, the decline in fibrinogen bearing EVs with age in this study reflects the lack of pro-coagulant and pro-inflammatory activity in these healthy individuals that may also play a protective role in preventing autoimmunity with aging. Consistent with the age-related alterations of plasma EVs during healthy aging, young plasma EVs induced stronger cell proliferation of human fibroblasts compared to old plasma EVs. Our findings provide a foundation for studying the phenotype and roles of these EV biomarkers in pathological aging and age-related diseases.

## Data availability statement

The original contributions presented in the study are included in the article/**Supplementary Material**. The datasets of EV proteomics for this study can be found in massive.ucsd.edu (Identifier: MSV000093511, Title “Plasma Extracellular Vesicle Proteomics in Healthy Donors”).

## Ethics statement

The studies involved human samples from a commercial vendor (Zen-bio acquired with IRB approval) and the completed Genetics of Generalized Osteoarthritis (GOGO) study that were approved by the Institutional Review Board (IRB) of Duke University. The studies were conducted in accordance with the local legislation and institutional requirements.

## Author contributions

XZ: Conceptualization, Data curation, Formal analysis, Funding acquisition, Investigation, Methodology, Project administration, Validation, Visualization, Writing – original draft, Writing – review & editing. SM: Data curation, Formal analysis, Investigation, Writing – review & editing. SN: Data curation, Formal analysis, Writing – review & editing. JH: Data curation, Investigation, Writing – review & editing. ES: Data curation, Formal analysis, Writing – review & editing. NA: Investigation, Writing – review & editing. CA: Supervision, Writing – review & editing. VK: Conceptualization, Data curation, Funding acquisition, Methodology, Project administration, Resources, Supervision, Writing – review & editing.
