# Supplementary material for: Immune system-related plasma extracellular vesicles in healthy aging
Source: Front Immunol. 2024 Apr 3;15:1355380. doi: 10.3389/fimmu.2024.1355380 (PMC11021711; doi:10.3389/fimmu.2024.1355380)
Supplement: Supplementary file 1 [file DataSheet_1.pdf]

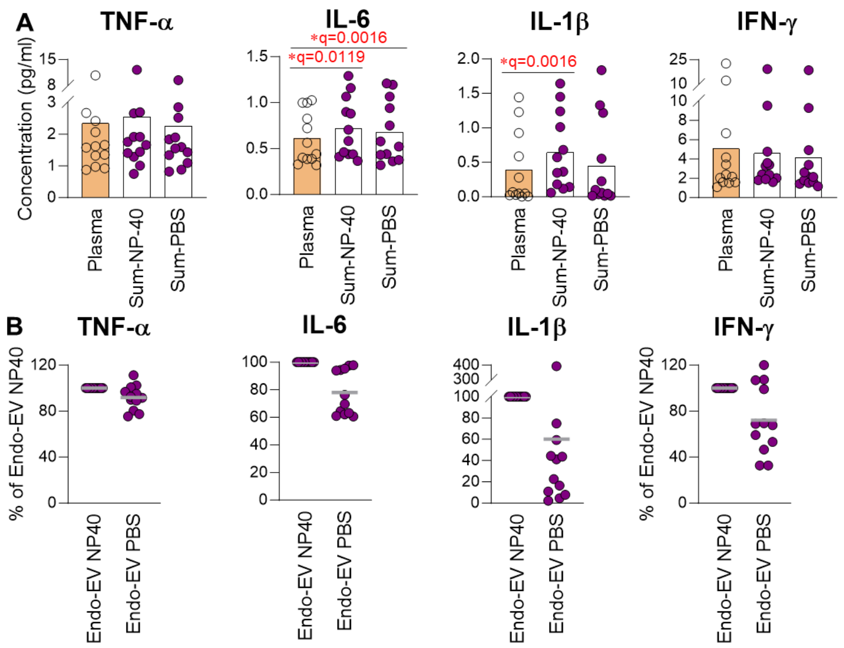

Supplementary Figure 1. MSD ELISA detected both exo-EV and endo-EV cytokines. Plasma EV pellets were separated from EV-depleted supernatants of healthy donors (n=12). EV pellets were lysed in NP40 lysis buffer or resuspended in double filtered PBS (df-PBS filtered by 100 nm filters) in the same volume as the EV-depleted supernatants. The concentrations of plasma exo-EV and endo-EV cytokines were measured by MSD multiplex ELISA whose sample diluent buffer contains 0.1-0.2% Triton-X 100. **(A)** Sum-NP40 represents the total summed concentrations of exo-EV cytokines and endo-EV cytokines yielded with the addition of NP40 lysis buffer. Sum-PBS represents the total summed concentrations of exo-EV cytokines and endo-EV cytokines yielded without the addition of NP40 lysis buffer but with df-PBS instead Comparisons were performed using Friedman test with Benjamini and Hochberg multiple comparisons; significant results were defined as FDR value ∗ q <0.05. **(B)** The graphs display the proportion of endo-EV cytokine concentrations as a ratio of the amount without additional NP40 to that with additional NP40. Each dot represents data of one study participant and the gray lines indicate the mean.
