# Supplementary material for: Immune system-related plasma extracellular vesicles in healthy aging
Source: Front Immunol. 2024 Apr 3;15:1355380. doi: 10.3389/fimmu.2024.1355380 (PMC11021711; doi:10.3389/fimmu.2024.1355380)
Supplement: Supplementary file 2 [file DataSheet_2.pdf]

Supplementary Table 1. Age-associated plasma EV peptides.

	Mixed Models with Age			Repeated Freeze-Thaw Cycle(s) vs. Baseline										
Peptide Name	P Value	Adjusted P Value	Coefficient	1 Time P Value	Adjusted P Value	3 Times P Value	Adjusted P Value	6 Times P Value	Adjusted P Value	Gene Name	Protein Name	Accession #	Peptide Sequence	Protein Position(s)
APOA1__19ox	0.0855	0.9996	9976	0.3711	1.0000	NA	NA	0.3711	1.0000	APOA1	Apolipoprotein A-I	P02647	EQLGPVTQEFWDNLEKETEGLRQEMSK	P02647 [86-112]
APOB__360	**0.0175**	0.9996	18826	0.3711	1.0000	NA	NA	NA	NA	APOB	Apolipoprotein B-100	P04114	TNPTGTQELLDIANYLMEQIQDDCTGDEDYT YLILR	P04114 [455-490]
CFH__119	0.0626	0.9996	-20477	0.1814	1.0000	1.0000	1.0000	1.0000	1.0000	CFH	Complement factor H	P08603	WSHPPSCIKTDCLSLPSFENAIPMGEK	P08603 [978-1004]
CFHR2__3	0.0626	0.9996	3279	0.3711	1.0000	NA	NA	1.0000	1.0000	CFHR2	Complement factor H-related protein 2	P36980	CLDPCVISQEIMEK	P36980 [203-216]
CLU__18	0.0988	0.9996	-3014	0.8551	1.0000	0.3613	1.0000	0.3613	1.0000	CLU	Clusterin	P10909	LRRELDESLQVAER	P10909 [323-336]
FGA__27	0.0937	0.9996	-26006	0.8551	1.0000	0.1003	1.0000	0.5839	1.0000	FGA	Fibrinogen alpha chain	P02671	GDFSSANNRDNTYNRVSEDLR	P02671 [115-135]
FGA__77	0.0723	0.9996	-906	NA	NA	NA	NA	NA	NA	FGA	Fibrinogen alpha chain	P02671	RLEVDIDIKIR	P02671 [168-178]
FGB__50	**0.0490**	0.9996	-1615837	0.3613	1.0000	0.3613	1.0000	0.5839	1.0000	FGB	Fibrinogen beta chain	P02675	REEAPSLRPAPPPISGGGYR	P02675 [53-72]
FGG__26	0.0985	0.9996	-36466	0.2012	1.0000	0.8551	1.0000	0.3613	1.0000	FGG	Fibrinogen gamma chain	P02679	IIPFNRLTIGEGQQHHLGGAK	P02679 [412-432]
FGG__64	0.0905	0.9996	-6696	0.5839	1.0000	0.1003	1.0000	1.0000	1.0000	FGG	Fibrinogen gamma chain	P02679	YLQEIYNSNNQKIVNLK	P02679 [135-151]
GC__50	**0.0273**	0.9996	12857	NA	NA	0.3711	1.0000	NA	NA	GC	Vitamin D-binding protein	P02774	SYLSMVGSCCTSASPTVCFLKER	P02774 [181-203]
GSN__18	0.0942	0.9996	-404	0.5839	1.0000	0.1003	1.0000	0.5839	1.0000	GSN	Gelsolin	P06396	GASQAGAPQGR	P06396 [33-43]
Ig_gamma1_heavy_chain__31	0.0870	0.9996	-22178	0.3613	1.0000	0.8551	1.0000	1.0000	1.0000	IGHG1	Immunoglobulin heavy constant gamma 1	P01857	TKPREEQYNSTYR	P01857 [172-184]; P0DOX5 [291-303]
Ig_lambda1_light_chain__6	**0.0436**	0.9996	-279213	0.8551	1.0000	1.0000	1.0000	0.1003	1.0000	IGLC1	Immunoglobulin lambda constant 1	P0CG04	ANPTVTLFPPSSEELQANK	B9A064 [113-131]; P0CG04 [5-23]; P0DOX8 [115-133]
KNG1__31	0.0907	0.9996	-2333	0.8551	1.0000	0.8551	1.0000	0.5839	1.0000	KNG1	Kininogen-1	P01042	RHDWGHEKQR	P01042 [428-437]
KRT5__2	0.0765	0.9996	38360	NA	NA	NA	NA	NA	NA	KRT5	Keratin, type II cytoskeletal 5	P13647	EYQELMNTK	P13647 [452-460]
KRT9__36	0.0582	0.9996	27285	NA	NA	NA	NA	NA	NA	KRT9	Keratin, type I cytoskeletal 9	P35527	VQALEEANNDLENK	P35527 [171-184]
LPA__32ox	0.0984	0.9996	9484	NA	NA	NA	NA	NA	NA	LPA	Apolipoprotein(a)	P08519	TPEYYPNAGLIMNYCR	P08519 [177-192]; [291-306]; [405-420]; [519-534]; [633-648]; [747-762]; [861-876]; [975-990]; [1089-1104]; [1203-1218]; [1317-1332]; [1431-1446]; [1545-1560]; [1659-1674]; [1773-1788]; [1887-1902]; [2001-2016]; [2115-2130]; [2229-2244]; [2343-2358]; [2457-2472]; [2571-2586]; [2685-2700]; [2799-2814]; [2913-2928]; [3027-3042]; [3141-3156]; [3255-3270]; [3369-3384]
MYH9__8	0.0901	0.9996	5647	NA	NA	NA	NA	NA	NA	MYH9	Myosin-9	P35579	ASITALEAK	P35579 [1807-1815]
ORM1__10	0.0936	0.9996	23701	1.0000	1.0000	1.0000	1.0000	1.0000	1.0000	ORM1	Alpha-1-acid glycoprotein 1	P02763	TYMLAFDVNDEKNWGLSVYADKPETTKEQL GEFYEALDCLR	P02763 [127-167]
QSOX1__10	0.0603	0.9996	24358	NA	NA	NA	NA	NA	NA	QSOX1	Sulfhydryl oxidase 1	O00391	SFYTAYLQR	O00391 [257-265]
SERPINA3__25	0.0947	0.9996	34920	0.3613	1.0000	0.5839	1.0000	0.8551	1.0000	SERPINA3	Alpha-1-antichymotrypsin	P01011	WVMVPMMSLHHLTIPYFRDEELSCTVVELK	P01011 [238-267]
SERPIND1__5	0.0986	0.9996	3379	1.0000	1.0000	1.0000	1.0000	0.3711	1.0000	SERPIND1	Heparin cofactor 2	P05546	FTVDRPFLFLIYEHRTSCLLFMGR	P05546 [469-492]
SHPK	0.0738	0.9996	981969	0.8551	1.0000	0.5839	1.0000	0.2012	1.0000	SHPK	Sedoheptulokinase	Q9UHJ6	SSGFPVHLLPDIAEPGSVAGR	Q9UHJ6 [219-239]
VWF__47	0.0675	0.9996	6351	NA	NA	NA	NA	NA	NA	VWF	von Willebrand factor	P04275	SKEFMEEVIQR	P04275 [1517-1527]
VWF__71	0.0727	0.9996	7150	NA	NA	NA	NA	NA	NA	VWF	von Willebrand factor	P04275	YTLFQIFSK	P04275 [1363-1371]

The table presents EV peptieds with p<0.1 from the mixed models with age.
NA indicates missing p values resulted from insufficient paris (n<2) of comparisons. The freeze-thaw analysis was only conducted when there were enough samples for the peptide in the two time points under consideration. i.e. at least two sets of repeated samples from the two time points
P values <0.05 are in bold.
