# Supplementary material for: Immune system-related plasma extracellular vesicles in healthy aging
Source: Front Immunol. 2024 Apr 3;15:1355380. doi: 10.3389/fimmu.2024.1355380 (PMC11021711; doi:10.3389/fimmu.2024.1355380)
Supplement: Supplementary file 3 [file DataSheet_3.pdf]

Supplementary Table 2. All FGA, FGB and FGG peptides detected in plasma of healthy donors.

Peptide Name	Gene Name	Peptide Sequence	Protein Name	Protein Position(s)
FGA__1	FGA	ALTDMPQMR	Fibrinogen alpha chain	P02671 [250-258]
FGA__1ox	FGA	ALTDMPQMR	Fibrinogen alpha chain	P02671 [250-258]
FGA__1ox	FGA	ALTDMPQMR	Fibrinogen alpha chain	P02671 [250-258]
FGA__2	FGA	AQLVDMK	Fibrinogen alpha chain	P02671 [161-167]
FGA__2ox	FGA	AQLVDMK	Fibrinogen alpha chain	P02671 [161-167]
FGA__3	FGA	AQLVDMKR	Fibrinogen alpha chain	P02671 [161-168]
FGA__3ox	FGA	AQLVDMKR	Fibrinogen alpha chain	P02671 [161-168]
FGA__4	FGA	DCDDVLQTHPSGTQSGIFNIK	Fibrinogen alpha chain	P02671 [631-651]
FGA__5	FGA	DLLPSRDRQHLPLIK	Fibrinogen alpha chain	P02671 [211-225]
FGA__6	FGA	DNTYNRVSEDLR	Fibrinogen alpha chain	P02671 [124-135]
FGA__7	FGA	DRQHLPLIK	Fibrinogen alpha chain	P02671 [217-225]
FGA__8	FGA	DSDWPFCSDEDWNYK	Fibrinogen alpha chain	P02671 [49-63]
FGA__9	FGA	DSDWPFCSDEDWNYKCPSGCR	Fibrinogen alpha chain	P02671 [49-69]
FGA__10	FGA	DSHSLTTNIMEILR	Fibrinogen alpha chain	P02671 [101-114]
FGA__10ox	FGA	DSHSLTTNIMEILR	Fibrinogen alpha chain	P02671 [101-114]
FGA__12	FGA	DYEDQQKQLEQVIAK	Fibrinogen alpha chain	P02671 [196-210]
FGA__13	FGA	EKVTSGSTTTTR	Fibrinogen alpha chain	P02671 [447-458]
FGA__14	FGA	EKVTSGSTTTTRR	Fibrinogen alpha chain	P02671 [447-459]
FGA__15	FGA	ESSSHHPGIAEFPSR	Fibrinogen alpha chain	P02671 [559-573]
FGA__16	FGA	ESSSHHPGIAEFPSRGK	Fibrinogen alpha chain	P02671 [559-575]
FGA__17	FGA	EVDLKDYEDQQK	Fibrinogen alpha chain	P02671 [191-202]
FGA__18	FGA	EVDLKDYEDQQKQLEQVIAK	Fibrinogen alpha chain	P02671 [191-210]
FGA__19	FGA	EVTKEVVTSEDGSDCPEAMDLGTLSGIGTLDGFR	Fibrinogen alpha chain	P02671 [477-510]
FGA__20	FGA	EVVTSEDGSDCPEAMDLGTLSGIGTLDGFR	Fibrinogen alpha chain	P02671 [481-510]
FGA__22	FGA	EVVTSEDGSDCPEAMDLGTLSGIGTLDGFRHRHPDEAAFFDTASTGK	Fibrinogen alpha chain	P02671 [481-527]
FGA__23	FGA	EYHTEKLVTSK	Fibrinogen alpha chain	P02671 [427-437]
FGA__24	FGA	EYHTEKLVTSKGDK	Fibrinogen alpha chain	P02671 [427-440]
FGA__25	FGA	GDFSSANNR	Fibrinogen alpha chain	P02671 [115-123]
FGA__26	FGA	GDFSSANNRDNTYNR	Fibrinogen alpha chain	P02671 [115-129]
FGA__27	FGA	GDFSSANNRDNTYNRVSEDLR	Fibrinogen alpha chain	P02671 [115-135]
FGA__28	FGA	GDSTFESK	Fibrinogen alpha chain	P02671 [592-599]
FGA__31	FGA	GGSTSYGTGSETESPR	Fibrinogen alpha chain	P02671 [272-287]
FGA__32	FGA	GGSTSYGTGSETESPRNPSSAGSWNSGSSGPGSTGNR	Fibrinogen alpha chain	P02671 [272-308]
FGA__33	FGA	GKSSSYSK	Fibrinogen alpha chain	P02671 [574-581]
FGA__34	FGA	GLIDEVNQDFTNR	Fibrinogen alpha chain	P02671 [72-84]
FGA__35	FGA	GLIDEVNQDFTNRINK	Fibrinogen alpha chain	P02671 [72-87]
FGA__36	FGA	GLIDEVNQDFTNRINKLK	Fibrinogen alpha chain	P02671 [72-89]
FGA__37	FGA	GSESGIFTNTK	Fibrinogen alpha chain	P02671 [548-558]
FGA__38	FGA	GSESGIFTNTKESSSHHPGIAEFPSR	Fibrinogen alpha chain	P02671 [548-573]
FGA__39	FGA	GSESGIFTNTKESSSHHPGIAEFPSRGK	Fibrinogen alpha chain	P02671 [548-575]
FGA__40	FGA	HPDEAAFFDTASTGK	Fibrinogen alpha chain	P02671 [513-527]
FGA__41	FGA	HQSACKDSDWPFCSDEDWNYK	Fibrinogen alpha chain	P02671 [43-63]
FGA__42	FGA	HQSACKDSDWPFCSDEDWNYKCPSGCR	Fibrinogen alpha chain	P02671 [43-69]
FGA__43	FGA	HRHPDEAAFFDTASTGK	Fibrinogen alpha chain	P02671 [511-527]
FGA__45	FGA	KVIEKVQHIQLLQK	Fibrinogen alpha chain	P02671 [144-157]
FGA__46	FGA	LEVDIDIK	Fibrinogen alpha chain	P02671 [169-176]
FGA__47	FGA	LKNSLFEYQK	Fibrinogen alpha chain	P02671 [88-97]
FGA__49	FGA	LVTSKGDKELR	Fibrinogen alpha chain	P02671 [433-443]
FGA__50	FGA	MADEAGSEADHEGTHSTK	Fibrinogen alpha chain	P02671 [603-620]
FGA__50ox	FGA	MADEAGSEADHEGTHSTK	Fibrinogen alpha chain	P02671 [603-620]
FGA__51	FGA	MADEAGSEADHEGTHSTKR	Fibrinogen alpha chain	P02671 [603-621]
FGA__51ox	FGA	MADEAGSEADHEGTHSTKR	Fibrinogen alpha chain	P02671 [603-621]
FGA__52	FGA	MADEAGSEADHEGTHSTKRGHAK	Fibrinogen alpha chain	P02671 [603-625]
FGA__52ox	FGA	MADEAGSEADHEGTHSTKRGHAK	Fibrinogen alpha chain	P02671 [603-625]
FGA__53	FGA	MELERPGGNEITR	Fibrinogen alpha chain	P02671 [259-271]
FGA__53ox	FGA	MELERPGGNEITR	Fibrinogen alpha chain	P02671 [259-271]
FGA__55	FGA	MKGLIDEVNQDFTNR	Fibrinogen alpha chain	P02671 [70-84]
FGA__55ox	FGA	MKGLIDEVNQDFTNR	Fibrinogen alpha chain	P02671 [70-84]
FGA__56	FGA	MKGLIDEVNQDFTNRINK	Fibrinogen alpha chain	P02671 [70-87]
FGA__56ox	FGA	MKGLIDEVNQDFTNRINK	Fibrinogen alpha chain	P02671 [70-87]
FGA__57	FGA	MKPVPDLVPGNFK	Fibrinogen alpha chain	P02671 [226-238]
FGA__57ox	FGA	MKPVPDLVPGNFK	Fibrinogen alpha chain	P02671 [226-238]
FGA__58	FGA	MKPVPDLVPGNFKSQLQK	Fibrinogen alpha chain	P02671 [226-243]
FGA__59	FGA	MKPVPDLVPGNFKSQLQKVPPEWK	Fibrinogen alpha chain	P02671 [226-249]
FGA__60	FGA	NNKDSHSLTTNIMEILR	Fibrinogen alpha chain	P02671 [98-114]

FGA__60ox	FGA	NNKDSHSLTTNIMEILR	Fibrinogen alpha chain	P02671 [98-114]
FGA__62	FGA	NPSSAGSWNSGSSGPGSTGNR	Fibrinogen alpha chain	P02671 [288-308]
FGA__63	FGA	NSLFEYQK	Fibrinogen alpha chain	P02671 [90-97]
FGA__65	FGA	NSLFEYQKNNKDSHSLTTNIMEILR	Fibrinogen alpha chain	P02671 [90-114]
FGA__66	FGA	QFTSSTSYNR	Fibrinogen alpha chain	P02671 [582-591]
FGA__67	FGA	QFTSSTSYNRGDSTFESK	Fibrinogen alpha chain	P02671 [582-599]
FGA__68	FGA	QFTSSTSYNRGDSTFESKSYK	Fibrinogen alpha chain	P02671 [582-602]
FGA__69	FGA	QHLPLIK	Fibrinogen alpha chain	P02671 [219-225]
FGA__70	FGA	QLEQVIAK	Fibrinogen alpha chain	P02671 [203-210]
FGA__71	FGA	QLEQVIAKDLLPSR	Fibrinogen alpha chain	P02671 [203-216]
FGA__72	FGA	QLEQVIAKDLLPSRDR	Fibrinogen alpha chain	P02671 [203-218]
FGA__73	FGA	REYHTEK	Fibrinogen alpha chain	P02671 [426-432]
FGA__74	FGA	REYHTEKLVTSK	Fibrinogen alpha chain	P02671 [426-437]
FGA__76	FGA	RLEVDIDIK	Fibrinogen alpha chain	P02671 [168-176]
FGA__77	FGA	RLEVDIDIKIR	Fibrinogen alpha chain	P02671 [168-178]
FGA__78	FGA	SQLQKVPPEWK	Fibrinogen alpha chain	P02671 [239-249]
FGA__80	FGA	SRIEVLK	Fibrinogen alpha chain	P02671 [136-142]
FGA__81	FGA	SRIEVLKR	Fibrinogen alpha chain	P02671 [136-143]
FGA__82	FGA	SRPVRDCDDVLQTHPSGTQSGIFNIK	Fibrinogen alpha chain	P02671 [626-651]
FGA__83	FGA	SSSYSKQFTSSTSYNR	Fibrinogen alpha chain	P02671 [576-591]
FGA__84	FGA	SSSYSKQFTSSTSYNRGDSTFESK	Fibrinogen alpha chain	P02671 [576-599]
FGA__85	FGA	SYKMADEAGSEADHEGTHSTK	Fibrinogen alpha chain	P02671 [600-620]
FGA__85ox	FGA	SYKMADEAGSEADHEGTHSTK	Fibrinogen alpha chain	P02671 [600-620]
FGA__86	FGA	SYKMADEAGSEADHEGTHSTKR	Fibrinogen alpha chain	P02671 [600-621]
FGA__86ox	FGA	SYKMADEAGSEADHEGTHSTKR	Fibrinogen alpha chain	P02671 [600-621]
FGA__87	FGA	TFPGFFSPMLGEFVSETESR	Fibrinogen alpha chain	P02671 [528-547]
FGA__87ox	FGA	TFPGFFSPMLGEFVSETESR	Fibrinogen alpha chain	P02671 [528-547]
FGA__88	FGA	TFPGFFSPMLGEFVSETESRGSESGIFTNTK	Fibrinogen alpha chain	P02671 [528-558]
FGA__88ox	FGA	TFPGFFSPMLGEFVSETESRGSESGIFTNTK	Fibrinogen alpha chain	P02671 [528-558]
FGA__89	FGA	TGKEKVTSGSTTTTR	Fibrinogen alpha chain	P02671 [444-458]
FGA__90	FGA	TVIGPDGHKEVTK	Fibrinogen alpha chain	P02671 [468-480]
FGA__91	FGA	TVIGPDGHKEVTKEVVTSEDGSDCPEAMDLGTLSGIGTLDGFR	Fibrinogen alpha chain	P02671 [468-510]
FGA__91ox	FGA	TVIGPDGHKEVTKEVVTSEDGSDCPEAMDLGTLSGIGTLDGFR	Fibrinogen alpha chain	P02671 [468-510]
FGA__93	FGA	TVTKTVIGPDGHKEVTK	Fibrinogen alpha chain	P02671 [464-480]
FGA__94	FGA	TWQDYKR	Fibrinogen alpha chain	P02671 [688-694]
FGA__95	FGA	VELEDWAGNEAYAEYHFR	Fibrinogen alpha chain	P02671 [726-743]
FGA__96	FGA	VIEKVQHIQLLQK	Fibrinogen alpha chain	P02671 [145-157]
FGA__98	FGA	VPPEWKALTDMPQMR	Fibrinogen alpha chain	P02671 [244-258]
FGA__99	FGA	VQHIQLLQK	Fibrinogen alpha chain	P02671 [149-157]
FGA__101	FGA	VSEDLRSR	Fibrinogen alpha chain	P02671 [130-137]
FGA__102	FGA	VTSGSTTTTR	Fibrinogen alpha chain	P02671 [449-458]
FGA__103	FGA	VVERHQSACKDSDWPFCSDEDWNYK	Fibrinogen alpha chain	P02671 [39-63]
FGB__1	FGB	AHYGGFTVQNEANK	Fibrinogen beta chain	P02675 [354-367]
FGB__2	FGB	AHYGGFTVQNEANKYQISVNK	Fibrinogen beta chain	P02675 [354-374]
FGB__3	FGB	AHYGGFTVQNEANKYQISVNKYR	Fibrinogen beta chain	P02675 [354-376]
FGB__4	FGB	APDAGGCLHADPDLGVLCPTGCQLQEALLQQERPIR	Fibrinogen beta chain	P02675 [89-124]
FGB__5	FGB	CHAANPNGR	Fibrinogen beta chain	P02675 [437-445]
FGB__6	FGB	DNDGWLTSDPR	Fibrinogen beta chain	P02675 [411-421]
FGB__7	FGB	DNDGWLTSDPRK	Fibrinogen beta chain	P02675 [411-422]
FGB__8	FGB	DNENVVNEYSSELEK	Fibrinogen beta chain	P02675 [164-178]
FGB__9	FGB	DNENVVNEYSSELEKHQLYIDETVNSNIPTNLR	Fibrinogen beta chain	P02675 [164-196]
FGB__10	FGB	ECEEIIR	Fibrinogen beta chain	P02675 [240-246]
FGB__11	FGB	ECEEIIRK	Fibrinogen beta chain	P02675 [240-247]
FGB__12	FGB	EDGGGWWYNR	Fibrinogen beta chain	P02675 [427-436]
FGB__13	FGB	EEAPSLRPAPPPISGGGYR	Fibrinogen beta chain	P02675 [54-72]
FGB__14	FGB	GGETSEMYLIQPDSSVKPYR	Fibrinogen beta chain	P02675 [248-267]
FGB__14ox	FGB	GGETSEMYLIQPDSSVKPYR	Fibrinogen beta chain	P02675 [248-267]
FGB__16	FGB	GHRPLDK	Fibrinogen beta chain	P02675 [45-51]
FGB__17	FGB	GHRPLDKK	Fibrinogen beta chain	P02675 [45-52]
FGB__18	FGB	GSWYSMR	Fibrinogen beta chain	P02675 [472-478]
FGB__18ox	FGB	GSWYSMR	Fibrinogen beta chain	P02675 [472-478]
FGB__73	FGB	GSWYSMRK	Fibrinogen beta chain	P02675 [472-479]
FGB__19	FGB	GTAGNALMDGASQLMGENR	Fibrinogen beta chain	P02675 [377-395]
FGB__20	FGB	HGTDDGVVWMNWK	Fibrinogen beta chain	P02675 [459-471]
FGB__20ox	FGB	HGTDDGVVWMNWK	Fibrinogen beta chain	P02675 [459-471]
FGB__22	FGB	HGTDDGVVWMNWKGSWYSMRK	Fibrinogen beta chain	P02675 [459-479]
FGB__23	FGB	HQLYIDETVNSNIPTNLR	Fibrinogen beta chain	P02675 [179-196]
FGB__24	FGB	IQKLESDVSAQMEYCR	Fibrinogen beta chain	P02675 [209-224]

FGB__24ox	FGB	IQKLESDVSAQMEYCR	Fibrinogen beta chain	P02675 [209-224]
FGB__25	FGB	IRPFFPQQ	Fibrinogen beta chain	P02675 [484-491]
FGB__26	FGB	KAPDAGGCLHADPDLGVLCPTGCQLQEALLQQERPIR	Fibrinogen beta chain	P02675 [88-124]
FGB__27	FGB	KGGETSEMYLIQPDSSVKPYR	Fibrinogen beta chain	P02675 [247-267]
FGB__27ox	FGB	KGGETSEMYLIQPDSSVKPYR	Fibrinogen beta chain	P02675 [247-267]
FGB__28	FGB	KGGETSEMYLIQPDSSVKPYRVYCDMNTENGGWTVIQNR	Fibrinogen beta chain	P02675 [247-285]
FGB__29	FGB	KQCSKEDGGGWWYNR	Fibrinogen beta chain	P02675 [422-436]
FGB__30	FGB	KREEAPSLRPAPPPISGGGYR	Fibrinogen beta chain	P02675 [52-72]
FGB__31	FGB	KWDPYKQGFGNVATNTDGK	Fibrinogen beta chain	P02675 [295-313]
FGB__32	FGB	LESDVSAQMEYCR	Fibrinogen beta chain	P02675 [212-224]
FGB__32ox	FGB	LESDVSAQMEYCR	Fibrinogen beta chain	P02675 [212-224]
FGB__33	FGB	MGPTELLIEMEDWK	Fibrinogen beta chain	P02675 [335-348]
FGB__33ox	FGB	MGPTELLIEMEDWK	Fibrinogen beta chain	P02675 [335-348]
FGB__34	FGB	MGPTELLIEMEDWKGDK	Fibrinogen beta chain	P02675 [335-351]
FGB__34ox	FGB	MGPTELLIEMEDWKGDK	Fibrinogen beta chain	P02675 [335-351]
FGB__35	FGB	MGPTELLIEMEDWKGDKVK	Fibrinogen beta chain	P02675 [335-353]
FGB__35ox	FGB	MGPTELLIEMEDWKGDKVK	Fibrinogen beta chain	P02675 [335-353]
FGB__35ox	FGB	MGPTELLIEMEDWKGDKVK	Fibrinogen beta chain	P02675 [335-353]
FGB__36	FGB	NSVDELNNNVEAVSQTSSSSFQYMYLLK	Fibrinogen beta chain	P02675 [125-152]
FGB__36ox	FGB	NSVDELNNNVEAVSQTSSSSFQYMYLLK	Fibrinogen beta chain	P02675 [125-152]
FGB__37	FGB	NSVDELNNNVEAVSQTSSSSFQYMYLLKDLWQK	Fibrinogen beta chain	P02675 [125-157]
FGB__38	FGB	NSVDELNNNVEAVSQTSSSSFQYMYLLKDLWQKR	Fibrinogen beta chain	P02675 [125-158]
FGB__39	FGB	NYCGLPGEYWLGNDK	Fibrinogen beta chain	P02675 [314-328]
FGB__40	FGB	NYCGLPGEYWLGNDKISQLTR	Fibrinogen beta chain	P02675 [314-334]
FGB__41	FGB	QCSKEDGGGWWYNR	Fibrinogen beta chain	P02675 [423-436]
FGB__74	FGB	QCSKEDGGGWWYNRCHAANPNGR	Fibrinogen beta chain	P02675 [423-445]
FGB__42	FGB	QDGSVDFGR	Fibrinogen beta chain	P02675 [286-294]
FGB__43	FGB	QDGSVDFGRK	Fibrinogen beta chain	P02675 [286-295]
FGB__44	FGB	QGFGNVATNTDGK	Fibrinogen beta chain	P02675 [301-313]
FGB__45	FGB	QGFGNVATNTDGKNYCGLPGEYWLGNDK	Fibrinogen beta chain	P02675 [301-328]
FGB__46	FGB	QGFGNVATNTDGKNYCGLPGEYWLGNDKISQLTR	Fibrinogen beta chain	P02675 [301-334]
FGB__47	FGB	QKQVKDNENVVNEYSSELEK	Fibrinogen beta chain	P02675 [159-178]
FGB__48	FGB	QVKDNENVVNEYSSELEK	Fibrinogen beta chain	P02675 [161-178]
FGB__49	FGB	QVKDNENVVNEYSSELEKHQLYIDETVNSNIPTNLR	Fibrinogen beta chain	P02675 [161-196]
FGB__50	FGB	REEAPSLRPAPPPISGGGYR	Fibrinogen beta chain	P02675 [53-72]
FGB__51	FGB	SILENLR	Fibrinogen beta chain	P02675 [200-206]
FGB__52	FGB	SKIQKLESDVSAQMEYCR	Fibrinogen beta chain	P02675 [207-224]
FGB__52ox	FGB	SKIQKLESDVSAQMEYCR	Fibrinogen beta chain	P02675 [207-224]
FGB__53	FGB	TMTIHNGMFFSTYDR	Fibrinogen beta chain	P02675 [396-410]
FGB__53ox	FGB	TMTIHNGMFFSTYDR	Fibrinogen beta chain	P02675 [396-410]
FGB__53ox	FGB	TMTIHNGMFFSTYDR	Fibrinogen beta chain	P02675 [396-410]
FGB__54	FGB	TMTIHNGMFFSTYDRDNDGWLTSDPR	Fibrinogen beta chain	P02675 [396-421]
FGB__54ox	FGB	TMTIHNGMFFSTYDRDNDGWLTSDPR	Fibrinogen beta chain	P02675 [396-421]
FGB__54ox	FGB	TMTIHNGMFFSTYDRDNDGWLTSDPR	Fibrinogen beta chain	P02675 [396-421]
FGB__55	FGB	TMTIHNGMFFSTYDRDNDGWLTSDPRK	Fibrinogen beta chain	P02675 [396-422]
FGB__55ox	FGB	TMTIHNGMFFSTYDRDNDGWLTSDPRK	Fibrinogen beta chain	P02675 [396-422]
FGB__56	FGB	TPCTVSCNIPVVSGK	Fibrinogen beta chain	P02675 [225-239]
FGB__57	FGB	TPCTVSCNIPVVSGKECEEIIR	Fibrinogen beta chain	P02675 [225-246]
FGB__58	FGB	TPCTVSCNIPVVSGKECEEIIRK	Fibrinogen beta chain	P02675 [225-247]
FGB__59	FGB	VKAHYGGFTVQNEANK	Fibrinogen beta chain	P02675 [352-367]
FGB__60	FGB	VKAHYGGFTVQNEANKYQISVNK	Fibrinogen beta chain	P02675 [352-374]
FGB__61	FGB	VYCDMNTENGGWTVIQNR	Fibrinogen beta chain	P02675 [268-285]
FGB__61ox	FGB	VYCDMNTENGGWTVIQNR	Fibrinogen beta chain	P02675 [268-285]
FGB__62	FGB	VYCDMNTENGGWTVIQNRQDGSVDFGR	Fibrinogen beta chain	P02675 [268-294]
FGB__64	FGB	WDPYKQGFGNVATNTDGK	Fibrinogen beta chain	P02675 [296-313]
FGB__65	FGB	WDPYKQGFGNVATNTDGKNYCGLPGEYWLGNDK	Fibrinogen beta chain	P02675 [296-328]
FGB__66	FGB	YQISVNK	Fibrinogen beta chain	P02675 [368-374]
FGB__67	FGB	YQISVNKYR	Fibrinogen beta chain	P02675 [368-376]
FGB__68	FGB	YYWGGQYTWDMAK	Fibrinogen beta chain	P02675 [446-458]
FGB__68ox	FGB	YYWGGQYTWDMAK	Fibrinogen beta chain	P02675 [446-458]
FGG__1	FGG	AIQLTYNPDESSKPNMIDAATLK	Fibrinogen gamma chain	P02679 [89-111]
FGG__1ox	FGG	AIQLTYNPDESSKPNMIDAATLK	Fibrinogen gamma chain	P02679 [89-111]
FGG__2	FGG	AIQLTYNPDESSKPNMIDAATLKSR	Fibrinogen gamma chain	P02679 [89-113]
FGG__3	FGG	ANQQFLVYCEIDGSGNGWTVFQK	Fibrinogen gamma chain	P02679 [200-222]
FGG__4	FGG	ANQQFLVYCEIDGSGNGWTVFQKR	Fibrinogen gamma chain	P02679 [200-223]
FGG__5	FGG	ASTPNGYDNGIIWATWK	Fibrinogen gamma chain	P02679 [383-399]
FGG__6	FGG	ASTPNGYDNGIIWATWKTR	Fibrinogen gamma chain	P02679 [383-401]
FGG__7	FGG	ASTPNGYDNGIIWATWKTRWYSMK	Fibrinogen gamma chain	P02679 [383-406]

FGG__8	FGG	CHAGHLNGVYYQGGTYSK	Fibrinogen gamma chain	P02679 [365-382]
FGG__9	FGG	DCQDIANK	Fibrinogen gamma chain	P02679 [178-185]
FGG__11	FGG	DNCCILDER	Fibrinogen gamma chain	P02679 [32-40]
FGG__12	FGG	DNCCILDERFGSYCPTTCGIADFLSTYQTK	Fibrinogen gamma chain	P02679 [32-61]
FGG__13	FGG	DTVQIHDITGK	Fibrinogen gamma chain	P02679 [167-177]
FGG__14	FGG	DTVQIHDITGKDCQDIANK	Fibrinogen gamma chain	P02679 [167-185]
FGG__16	FGG	EGFGHLSPTGTTEFWLGNEK	Fibrinogen gamma chain	P02679 [239-258]
FGG__17	FGG	EGFGHLSPTGTTEFWLGNEKIHLISTQSAIPYALR	Fibrinogen gamma chain	P02679 [239-273]
FGG__18	FGG	EGFGHLSPTGTTEFWLGNEKIHLISTQSAIPYALRVELEDWNGR	Fibrinogen gamma chain	P02679 [239-282]
FGG__19	FGG	EKVAQLEAQCQEPCKDTVQIHDITGK	Fibrinogen gamma chain	P02679 [152-177]
FGG__20	FGG	FFTSHNGMQFSTWDNDNDKFEGNCAEQDGSGWWMNK	Fibrinogen gamma chain	P02679 [329-364]
FGG__21	FGG	FGSYCPTTCGIADFLSTYQTK	Fibrinogen gamma chain	P02679 [41-61]
FGG__22	FGG	FGSYCPTTCGIADFLSTYQTKVDK	Fibrinogen gamma chain	P02679 [41-64]
FGG__23	FGG	IHLISTQSAIPYALR	Fibrinogen gamma chain	P02679 [259-273]
FGG__24	FGG	IHLISTQSAIPYALRVELEDWNGR	Fibrinogen gamma chain	P02679 [259-282]
FGG__25	FGG	IHLISTQSAIPYALRVELEDWNGRTSTADYAMFK	Fibrinogen gamma chain	P02679 [259-292]
FGG__26	FGG	IIPFNRLTIGEGQQHHLGGAK	Fibrinogen gamma chain	P02679 [412-432]
FGG__27	FGG	IVNLKEK	Fibrinogen gamma chain	P02679 [147-153]
FGG__28	FGG	KMLEEIMK	Fibrinogen gamma chain	P02679 [114-121]
FGG__28ox	FGG	KMLEEIMK	Fibrinogen gamma chain	P02679 [114-121]
FGG__28ox	FGG	KMLEEIMK	Fibrinogen gamma chain	P02679 [114-121]
FGG__29	FGG	KMLEEIMKYEASILTHDSSIR	Fibrinogen gamma chain	P02679 [114-134]
FGG__29ox	FGG	KMLEEIMKYEASILTHDSSIR	Fibrinogen gamma chain	P02679 [114-134]
FGG__29ox	FGG	KMLEEIMKYEASILTHDSSIR	Fibrinogen gamma chain	P02679 [114-134]
FGG__30	FGG	KNWIQYK	Fibrinogen gamma chain	P02679 [232-238]
FGG__31	FGG	KNWIQYKEGFGHLSPTGTTEFWLGNEK	Fibrinogen gamma chain	P02679 [232-258]
FGG__32	FGG	LDGSVDFK	Fibrinogen gamma chain	P02679 [224-231]
FGG__33	FGG	LDGSVDFKK	Fibrinogen gamma chain	P02679 [224-232]
FGG__34	FGG	LDGSVDFKKNWIQYK	Fibrinogen gamma chain	P02679 [224-238]
FGG__35	FGG	LTIGEGQQHHLGGAK	Fibrinogen gamma chain	P02679 [418-432]
FGG__36	FGG	LTYAYFAGGDAGDAFDGFDFGDDPSDK	Fibrinogen gamma chain	P02679 [302-328]
FGG__37	FGG	MLEEIMKYEASILTHDSSIR	Fibrinogen gamma chain	P02679 [115-134]
FGG__37ox	FGG	MLEEIMKYEASILTHDSSIR	Fibrinogen gamma chain	P02679 [115-134]
FGG__38	FGG	MLEEIMKYEASILTHDSSIRYLQEIYNSNNQK	Fibrinogen gamma chain	P02679 [115-146]
FGG__39	FGG	NWIQYKEGFGHLSPTGTTEFWLGNEK	Fibrinogen gamma chain	P02679 [233-258]
FGG__42	FGG	QSGLYFIKPLK	Fibrinogen gamma chain	P02679 [189-199]
FGG__43	FGG	QSGLYFIKPLKANQQFLVYCEIDGSGNGWTVFQK	Fibrinogen gamma chain	P02679 [189-222]
FGG__44	FGG	QSGLYFIKPLKANQQFLVYCEIDGSGNGWTVFQKR	Fibrinogen gamma chain	P02679 [189-223]
FGG__45	FGG	QVRPEHPAETEYDSLYPEDDL	Fibrinogen gamma chain	P02679 [433-453]
FGG__46	FGG	RLDGSVDFK	Fibrinogen gamma chain	P02679 [223-231]
FGG__47	FGG	RLDGSVDFKK	Fibrinogen gamma chain	P02679 [223-232]
FGG__48	FGG	TRWYSMK	Fibrinogen gamma chain	P02679 [400-406]
FGG__50	FGG	TSEVKQLIK	Fibrinogen gamma chain	P02679 [80-88]
FGG__51	FGG	TSTADYAMFK	Fibrinogen gamma chain	P02679 [283-292]
FGG__51ox	FGG	TSTADYAMFK	Fibrinogen gamma chain	P02679 [283-292]
FGG__52	FGG	TSTADYAMFKVGPEADKYR	Fibrinogen gamma chain	P02679 [283-301]
FGG__53	FGG	VAQLEAQCQEPCK	Fibrinogen gamma chain	P02679 [154-166]
FGG__54	FGG	VAQLEAQCQEPCKDTVQIHDITGK	Fibrinogen gamma chain	P02679 [154-177]
FGG__55	FGG	VAQLEAQCQEPCKDTVQIHDITGKDCQDIANK	Fibrinogen gamma chain	P02679 [154-185]
FGG__56	FGG	VDKDLQSLEDILHQVENK	Fibrinogen gamma chain	P02679 [62-79]
FGG__57	FGG	VDKDLQSLEDILHQVENKTSEVK	Fibrinogen gamma chain	P02679 [62-84]
FGG__58	FGG	VELEDWNGR	Fibrinogen gamma chain	P02679 [274-282]
FGG__59	FGG	VELEDWNGRTSTADYAMFK	Fibrinogen gamma chain	P02679 [274-292]
FGG__60	FGG	VGPEADKYR	Fibrinogen gamma chain	P02679 [293-301]
FGG__62	FGG	YEASILTHDSSIR	Fibrinogen gamma chain	P02679 [122-134]
FGG__63	FGG	YLQEIYNSNNQK	Fibrinogen gamma chain	P02679 [135-146]
FGG__64	FGG	YLQEIYNSNNQKIVNLK	Fibrinogen gamma chain	P02679 [135-151]
FGG__65	FGG	YLQEIYNSNNQKIVNLKEK	Fibrinogen gamma chain	P02679 [135-153]
